# Hemodynamic Performance of Dysfunctional Prosthetic Heart Valve with the Concomitant Presence of Subaortic Stenosis: In Silico Study

**DOI:** 10.3390/bioengineering7030090

**Published:** 2020-08-07

**Authors:** Othman Smadi, Anas Abdelkarim, Samer Awad, Thakir D. Almomani

**Affiliations:** 1 Department of Biomedical Engineering, College of Engineering, The Hashemite University, Zarqa 13133, Jordan; samer.awad@hu.edu.jo (S.A.); thakir2000@hu.edu.jo (T.D.A.); 2 Institute of Automatic Control, Faculty of Electrical and Computer Engineering, Technische Universität Kaiserslautern, 67663 Kaiserslautern, Germany; abelkari@rhrk.uni-kl.de

**Keywords:** prosthetic heart valves, CFD, doppler echocardiography, subaortic stenosis, heart valve diseases, cardiovascular diseases

## Abstract

The prosthetic heart valve is vulnerable to dysfunction after surgery, thus a frequent assessment is required. Doppler electrocardiography and its quantitative parameters are commonly used to assess the performance of the prosthetic heart valves and provide detailed information on the interaction between the heart chambers and related prosthetic valves, allowing early detection of complications. However, in the case of the presence of subaortic stenosis, the accuracy of Doppler has not been fully investigated in previous studies and guidelines. Therefore, it is important to evaluate the accuracy of the parameters in such cases to get early detection, and a proper treatment plan for the patient, at the right time. In the current study, a CFD simulation was performed for the blood flow through a Bileaflet Mechanical Heart Valve (BMHV) with concomitant obstruction in the Left Ventricle Outflow Tract (LVOT). The current study explores the impact of the presence of the subaortic on flow patterns. It also investigates the accuracy of (BMHV) evaluation using Doppler parameters, as proposed in the American Society of Echocardiography (ASE) guidelines.

## 1. Introduction

Subaortic stenosis (SAS) is a narrowing in the functional valve area, causing a significant obstruction to the blood flow through the Left Ventricular Outflow Tract (LVOT). Consequently, the outflow gradient across the valve becomes higher [1,2,3]. Most SAS is due to congenital heart valve diseases, while it could be due to acquired heart valve disease [4,5].

According to the shape of obstruction, the SAS is classified into two types. The first type is discrete (membranous) subaortic stenosis that is defined as a fibrous membrane extending across the anterior portion of the LVOT. The second type of SAS is tunnel-shaped and may occur due to muscular hypertrophy of the interventricular septum. The existence of SAS, in general, might add an extra load onto the left heart ventricle, which increases the risk of sudden death from ventricular fibrillation [6]. SAS can be also categorized as fixed and dynamic SAS, based on the obstacle’s dimensions, and whether stable or varied [7,8]. In most cases, the dimensions of the obstructive orifice are static from beat to beat, which is referred to as fixed SAS, while in the dynamic type the severity of obstruction is changed by heart rate, and also usually alters as the systole progresses [9,10].

SAS might lead to aortic valve incompetency, stenotic aortic valve, and infective endocarditis [11]. Thus, surgery is required for treatment. Moreover, in aortic valve stenosis or insufficiency situations that exceed the mild type, the aortic valve is replaced by a prosthetic valve in the same surgery [12,13]. The prosthetic valve could be a biological heart valve or mechanical heart valve. The mechanical heart valve is the favorable option for patients who are under 65 years old, because of durability. However, it is more thrombogenic, thus the patients must take lifelong anticoagulants [14,15].

The recurrence of SAS after surgical correction has a high rate, of 37%. Besides, the opening and closure of the prosthetic heart valve might be restricted because of pannus formation and/or thrombosis formation, leading to both prosthetic valve dysfunction and stenosis. Pannus formation is a proliferation of fibro-elastic tissue and collagen due to the body’s inflammatory reaction to the valve prosthesis, which occurs in the tissue–valve interface and creeps along the suture lines, leading to prosthetic valve stenosis or SAS in the LVOT [16,17]. The average rate of prosthetic heart valve dysfunction ranges between 10% and 30% at 10 years of implantation [18].

In order to avoid the risk of complications after valve replacement, frequent assessment for the early detection of complications is essential. Different techniques of medical imaging modalities are used in diagnoses, such as Magnetic Resonance Imaging (MRI), Computed Tomography (CT) or Doppler echocardiography. Doppler echocardiography is a common option for preliminary assessment due to its features of availability, non-invasiveness, and being radiation-free and cost-effective. Different quantitative parameters are being used for the evaluation of prosthetic valves, including trans-prosthetic velocity and gradient, effective orifice area (EOA) and the Doppler velocity index (DVI). Moreover, Doppler parameters could be used to evaluate the presence and severity of subaortic stenosis [19,20].

Various numerical studies of BMHVs focused on normally functioning as well as dysfunctional BMHVs, with an emphasis on the velocity field, vortex dynamics, turbulence characteristics, leaflets dynamics, and the hinge mechanism and its relationship with platelet activation and/or hemolysis. Some of these studies did not consider the opening and closing phases of BMHV (fixed leaflets), and simulated the flow for the fully open position with different inlet flow conditions, which ranged from steady state flow conditions [21,22,23] to unsteady state (pulsatile) flow conditions [24,25,26,27]. Moreover, the mobility of BMHV leaflets was simulated using Fluid Solid Interaction (FSI) methods. However, the nature of flow in many FSI studies was considered to be laminar, and in some cases the weak coupling method in FSI was adopted (leaflets mobility was assigned) [28,29,30,31,32].

Using cardiac simulators, a set of in vitro studies investigated blood flow behavior downstream of BMHV in terms of level of turbulence, shear stress, and coherent structure. Clinically related echo Doppler parameters, such as EOA and Transvalvular pressure gradient, were also explored [33,34,35,36].

In the current study, a CFD simulation was performed for the blood flow through the Bileaflet Mechanical Heart Valve (BMHV) with concomitant presence of LVOT obstruction (tunnel shape). Different scenarios of dysfunction severity of BMHV, with the presence of different levels of LVOT obstruction, were introduced. The impact of the presence of subaortic stenosis on BMHV performance, and the accuracy of the diagnosis of Echo Doppler parameters as proposed in ASE guidelines, were explored [37].

## 2. Materials and Methods

### 2.1. Numerical Simulation Setup

In this study, a 2-D model based on a 27 mm St. Jude Medical BMHV was created to study the flow through normal and dysfunctional BMHV. The leaflet position was examined in three situations: a fully opened position (normal function), an intermediate position (50% one leaflet dysfunction) and a fully closed position (100% one leaflet dysfunction) with one intermediate position (50% one leaflet dysfunction). Furthermore, SAS (tunnel shape) was examined at five different hydraulic inlet diameters, from 27 mm to 19 mm (50% maximum area reduction), with 2 mm reductions in each step. Combining leaflet position situations and SAS cases, we have 15 models in total.

**Figure 1:**
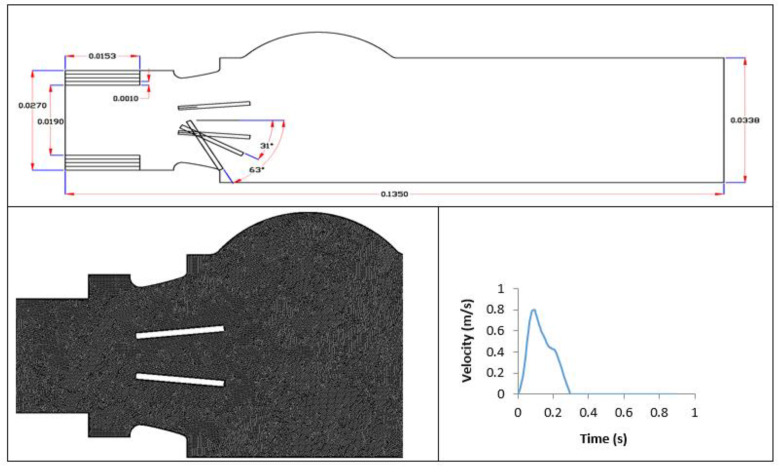
Model geometry with dimensions (m) for 15 different cases: 0% dysfunction (LVOT diameter: 27, 25, 23, 21 and 19 mm); 50% dysfunction (LVOT diameter: 27, 25, 23, 21 and 19 mm) and 100% dysfunction (LVOT diameter: 27, 25, 23, 21 and 19 mm). Mesh quality and velocity file for the healthy model at normal cardiac output (5 L/min) are also shown.

All models were drawn using AutoCAD software and simulated by FLUENT-ANSYS 14.5. The mesh generator tool is used to create high-quality triangle mesh geometry with, on average, 200,000 elements for each model (Figure 1). The flow fluid analysis system tool (Fluent) based on a finite volume method was used to run the numerical simulation.

BMHV has been chosen in this study since it is the most frequently implanted prosthetic heart valve. The current study focused on the forward macro-scale flow characteristics downstream of BMHV, and therefore the microcirculation of hinge housing was not simulated, and the forward flow during the valve fully open period (From 45 to 250 ms) was investigated [27,38]. However, despite omitting the opening and closing phases of the BMHV in the simulation, good agreement was found between numerical simulations and in vitro studies in terms of velocity profiles and magnitudes, recirculation zones, vortex shedding mechanism and echo Doppler parameters [21,39,40].

The simulation was performed under pulsatile conditions with an experimental pulsatile flow as inlet condition, as shown in Figure 1, which was adjustable to different SAS severity and cardiac outputs. In addition, the outlet condition was at the ambient pressure, for all cases, and a no-slip condition at the walls was adopted. In this study, the heart rate for all cases was constant at 70 bpm (systolic phase duration 0.3 s) while the mean cardiac outputs were varied from 3 L/min, to 5 L/min to 7 L/min, to examine the impact of low, normal and high cardiac output. Therefore, 45 cases were simulated (15 cases for each cardiac output).

In terms of blood properties, the values of density and dynamic viscosity were considered as 1060 kg/m^3^ and 0.0035 Pa.s, respectively. The blood was considered a Newtonian fluid which is reasonable for blood flow in large arteries such as the aorta and through mechanical heart valves [41].

Inlet conditions corresponded to Remax = 4203–13,945; Reaverage = 713–2672 and Womersley number (α) = 16.2.

A standard k−ω turbulent flow model with 5% turbulent intensity was used to simulate the flow during the full cardiac cycle [42]. The time step was chosen to be 0.5 ms to achieve time-step independence and a maximum of 30 iterations for each time step. The results were extracted for the fifth cycle of simulation as the periodicity was reached at this cycle.

Clinically, the BMHV’s performance was evaluated according to the recommendations of the American Society of Echocardiography’s Guidelines [37]. The studied hemodynamic parameters included: the maximum velocity magnitude through the valve, which is called the transvalvular peak velocity (Vpeak); mean transvalvular pressure gradient (TPGmean), which represents the average pressure drop across the valve and is determined based on the average of pressure drop over the systolic phase, where TPG is calculated based on the simplified Bernoulli equation (TPG$=V^{2})$; Doppler velocity index (DVI), which is calculated based on the ratio of the peak velocity of the left ventricle outflow track (V_LVOT) to the transvalvular peak velocity (DVI = V_LVOT/Vpeak) and effective orifice area (EOA) that represent the physically available area of the BMHV, and this is the minimum cross-sectional area (called vena contracta) of flow jet when maximum velocity is reached downstream of the valve, calculated based on the ratio of volume flow rate (Q) to the transvalvular peak velocity (EOA = Q/Vpeak).

### 2.2. Validation

**Figure 2:**
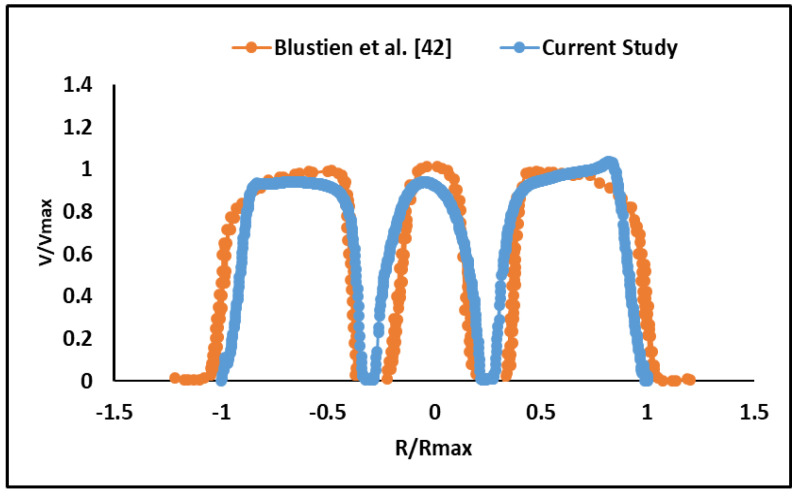
Comparison between normalized velocity of the current study and the study of Bluestein and co-authors around the peak systole. Velocity profile was measured downstream of the BMHV and near the trailing edges of the leaflets. (R/Rmax) is the normalized radius.

The current CFD model for blood flow through a normal BMHV around the peak systole was validated against the numerical and experimental study by Bluestein and co-workers [39]. The velocity profile was captured downstream of the valve and near the trailing edge of leaflets, and the plotted velocity and positions were normalized. (R/Rmax) as the normalized ratio represents radius over maximum radius. A good agreement was found between the two studies as shown in Figure 2.

## 3. Results

### 3.1. Hemodynamics

#### 3.1.1. Velocity Contours

**Figure 3:**
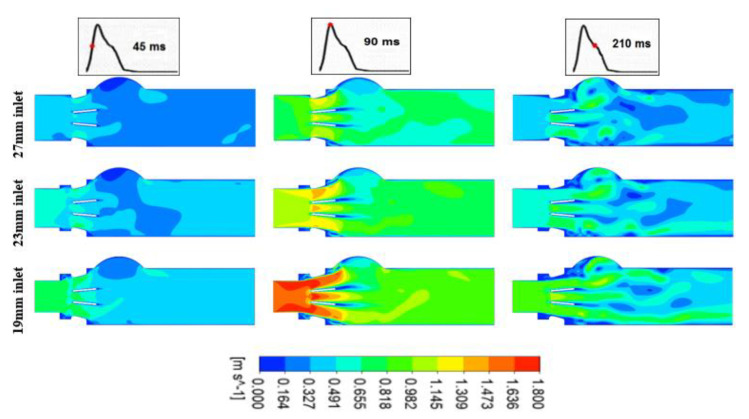
Velocity contours at three LVOT diameters (27, 23 and 19 m) at three different time instants (45 (acceleration phase), 90 (peak) and 210 ms (deceleration phase)) during the systole for normal BMHV at normal cardiac output (5 L/min).

**Figure 4:**
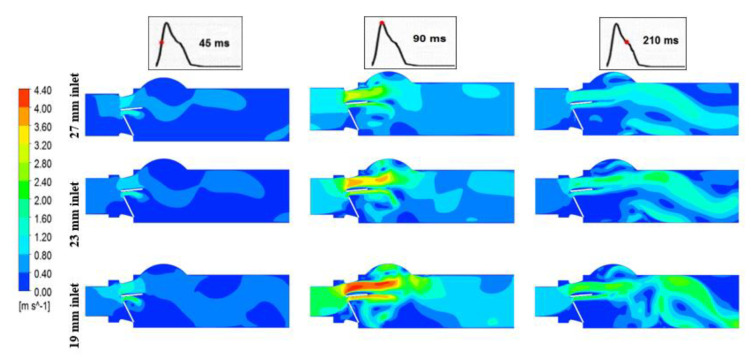
Velocity contours for three LVOT diameters (27, 23 and 19 m) at three different time instants (45 (acceleration phase), 90 ms (peak) and 210 ms (deceleration phase)) during the systole for 100% dysfunctional BMHV at the normal cardiac output (5 L/min).

Figure 3 and Figure 4 below show velocity contours at the normal cardiac output (5 L/min) for the entire field, at three different time instants (45 (acceleration phase), 90 (peak) and 210 ms (deceleration phase)) during the systole, for both a normal and a 100% dysfunctional leaflet at different LVOT diameters (27, 23 and 19 mm), respectively.

Figure 3 shows the flow rate through the normal valve. The flow patterns were uniform at the valve orifices (one central and two laterals). On the other hand, in Figure 4, with the presence of the 100% dysfunctional leaflet, the flow direction changed to be mainly lateral through the other normally functioning leaflet.

The peak velocity magnitude was proportional to the severity of both subaortic stenosis and valve dysfunction. As shown in Figure 3, peak velocity for a normally functioning valve, with the presence of only severe subaortic stenosis (19 mm LVOT diameter), was increased from 1.36 m/s to 1.81 m/s (+33%). Similarly, as shown in Figure 4, peak velocity for 100% leaflet dysfunction with the concomitant presence of severe subaortic stenosis (19 mm LVOT diameter) was increased from 3.14 m/s to 4.37 m/s (+39%). Furthermore, by comparing between the healthy model (no valve dysfunction nor sub-valve stenosis) and the most severe model (100% leaflet dysfunction and severe subaortic stenosis (19 mm inlet)), the Vpeak increased by 221% from 1.36 m/s to 4.37 m/s.

**Figure 5:**
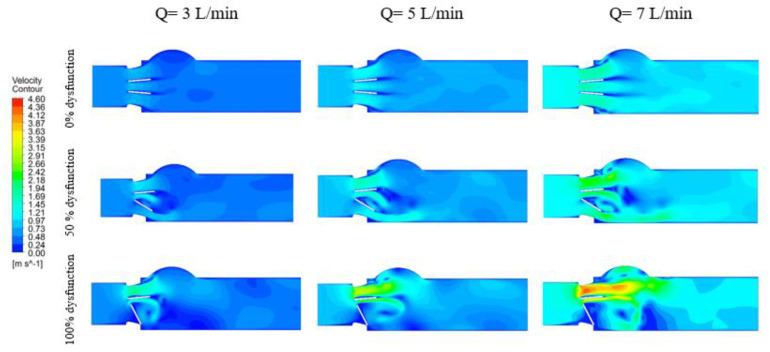
Velocity contours at peak systole (90 ms) for different percentages of BMHV dysfunction at three different cardiac outputs (3, 5 and 7 L/min) for 27 mm LVOT.

**Figure 6:**
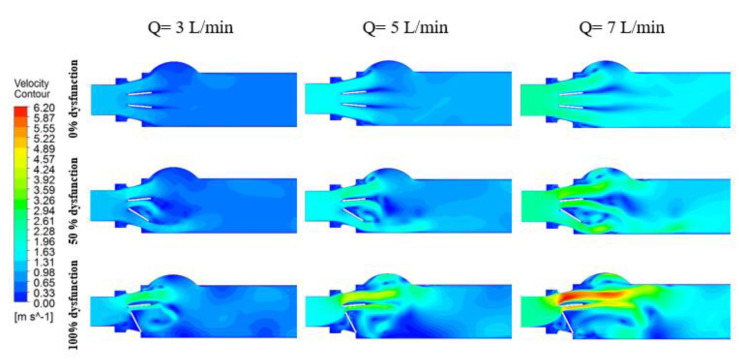
Velocity contours at peak systole (90 ms) for different percentages of BMHV dysfunction at three different cardiac outputs (3, 5 and 7 L/min) for 19 mm LVOT.

Figure 5 and Figure 6 show the velocity contours at peak systole for different percentages of BMHV dysfunction at three different cardiac outputs (3, 5 and 7 L/min) for 27 and 19 mm LVOT diameter.

The peak velocity magnitude was proportional to the cardiac output. In the case of the healthy model, the velocity increased from 0.86 m/s (at Q = 3 m/s) to 1.98 m/s (Q = 7 m/s), a 130% increase. Meanwhile, with the presence of severe subaortic stenosis and severe valve dysfunction (Figure 5), the peak velocity increased from 2.97 m/s to 6.20 m/s, at flow rates equal to 3 and 7 L/min, respectively (+109%).

It is worth noting that, in both figures, the distribution of velocity jets and the location of the recirculation zones was not altered by the magnitude of cardiac output. However, introducing the valve dysfunction forced the flow to be more lateral, which in turn affected the pattern of vortex shedding. Furthermore, introducing the LVOT obstruction created a large recirculation zone behind the dysfunctional leaflet.

#### 3.1.2. Vortex Dynamics

**Figure 7:**
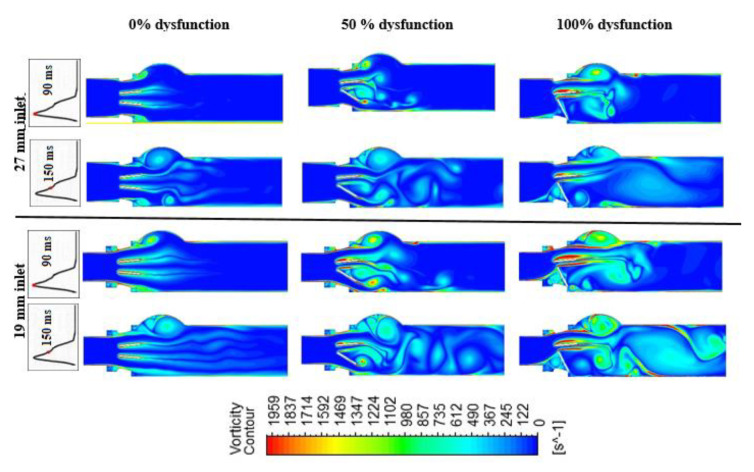
Vorticity distributions downstream of a healthy and dysfunction heart valve for both 27 mm and 19 mm inlet diameter at Q = 7 L/min and two time instances: peak time (90 ms) and during deceleration time (150 ms).

Figure 7 shows the vortex formation for a healthy and dysfunctional BMHV, for both 27 mm and 19 mm LVOT diameters at Q = 7 L/min, and at two different time instants (90 ms (peak systole) and 150 ms (deceleration phase of systole)). The vortex structure was dependent on the severity of both valve dysfunction and subaortic stenosis. One dominant vortex was formed in the sinus area in the case of the healthy valve [43]. The vortex shedding mechanism (Von Karman vortex street) was observed in all healthy models, and the strength was proportional to the severity of subaortic stenosis [39,44]. On the other hand, in the case of 100% leaflet dysfunction, many vortices were formed, not only downstream of the valve but a vortex structure upstream from the valve was also formed. Moreover, compared to the deceleration period, the vortex structures at the peak time (90 ms) had higher amplitudes, while during the deceleration period the vortices were larger in size and number and proceeded more toward the aorta.

In the case of 50% leaflet dysfunction, the flow behavior was dramatically changed and different vortical structures were formed; one of them inside the sinus region, and the others formed between and in the wake of the valve leaflets. The 50% dysfunctional leaflet models showed more complex vortical flow downstream of the valve with multiple small and large-scale vortices compared, to both the normal and 100% dysfunctional leaflet. The impact of the severity of subaortic stenosis was significant regardless of the severity of leaflet dysfunction. The strength, the covered region and the number of vortical structures were all significantly increased by the presence of subaortic stenosis.

The vorticity magnitude was proportional to cardiac output and the level of LVOT obstruction. However, the vortex shedding mechanism at the trailing edge of the BMHV, and the location of the recirculation zones in the sinus area and downstream of the valve, were not altered by the cardiac output magnitude.

#### 3.1.3. Turbulent Shear Stress

**Figure 8:**
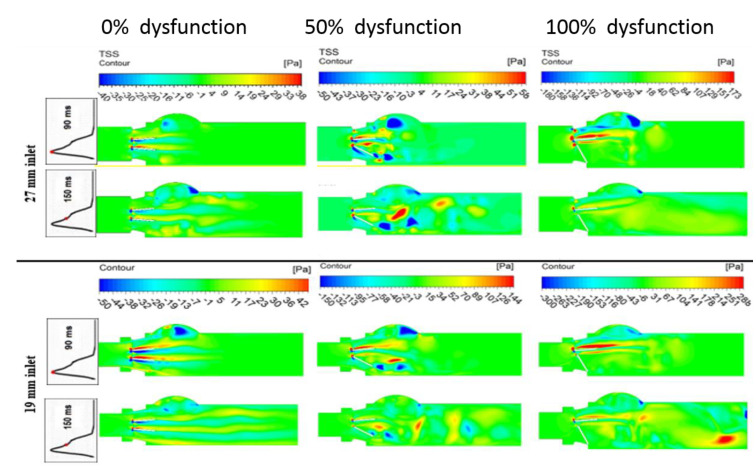
Shear stress magnitude contours of a healthy and dysfunction heart valve for both 27 mm and 19 mm inlet diameter at Q = 7 L/min and two time instances: peak time (90 ms) and during deceleration time (150 ms).

Figure 8 shows the contours of the Turbulent Shear Stress (TSS) magnitude for the healthy and dysfunctional BMHV, for both the 27 mm and 19 mm LVOT diameters at Q = 7 L/min and two different time instances: peak systole (90 ms) and during the deceleration phase (150 ms). The maximum magnitude (288 Pa) of TSS was observed when the leaflet was completely dysfunctional and the LVOT diameter was reduced to 19 mm. In the healthy model, the relatively high TSS magnitude was at its highest between and in the wake of the two leaflets, and close to the trailing edge of each leaflet. In the meantime, in the case of the partially dysfunctional leaflet, the dominant regions of TSS started to be moved to the lower side of the valve, as well as into the wake of the dysfunctional leaflet and adjacent to the sinus region of the lower wall. However, when the dysfunction of the leaflet reached 100%, the shear stress had its highest value around the upper side of the normal leaflet and around the sinus region of the upper wall. Moreover, the subaortic stenosis only elevated the magnitude of TSS, without significant changes in the location and the pattern of TSS in the entire field.

### 3.2. Doppler Echocardiographic Measurements

**Figure 9:**
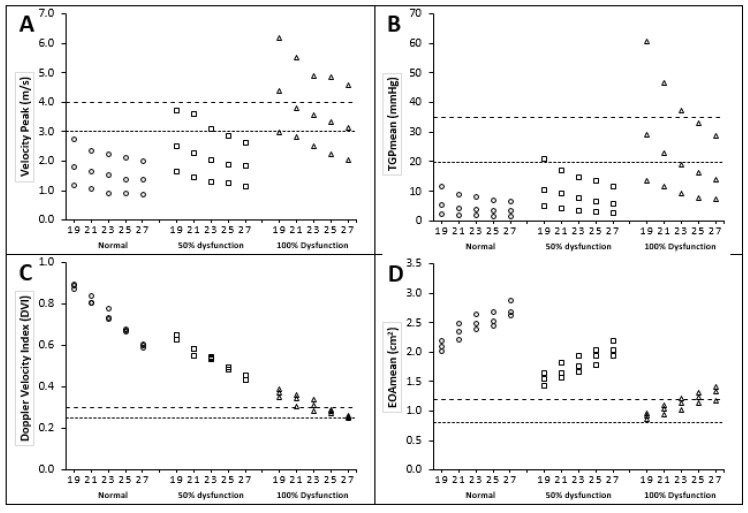
The Doppler echocardiographic parameters calculated for the (0, 50, 100%) of valve dysfunction with the presence of different sub valvar stenosis at three flow rates. (**A**) Peak velocity; (**B**) mean transvalvular pressure gradient; (**C**) Doppler velocity index (DVI); and (**D**) effective orifice area (EOA). The dashed and dotted lines represent the lower and upper proposed cut-off values in the ASE guidelines, respectively.

The cases of BMHV dysfunction regarding limited motion of only one leaflet were shown, by Montorsi and coauthors, as the most difficult to recognize with the clinical diagnosis protocol [30]. Therefore, we focused mainly on demonstrating and investigating the flow patterns for one dysfunctional leaflet only. Figure 9 and Table 1 show the data of Vpeak, TPGmean, DVI and valve EOA as a function of LVOT diameter and level of leaflet dysfunction, whereby 0%, 50% and 100% restriction of the opening of one leaflet correspond to no, mild (25% restriction in total valve area) and moderate-to-severe valve (50% restriction in total valve area) dysfunction, respectively. Moreover, Table 2 shows the sensitivity and specificity of the Doppler parameters with and without considering the cases of LVOT obstruction.

#### 3.2.1. Peak Velocity and Mean Transvalvular Pressure Gradient

Figure 9A shows the relationship between LVOT diameter, percentage of leaflet dysfunction and peak velocity magnitude (Vpeak). It was strongly flow-dependent, and proportional to flow rate for all cases (the higher the flow rate, the higher the Vpeak). Further, Vpeak was strongly proportional to the severity of subaortic stenosis for all cases (the smaller the LVOT diameter, the higher the Vpeak). For all cases of normally functioning BMHVs, Vpeak did not exceed the peak velocity magnitude of 3 m/s regardless of subaortic stenosis severity (the suggested ASE threshold for possible dysfunction). Meanwhile, the peak velocity for a few cases of 50% leaflet dysfunction exceeded the 3 m/s threshold (20%). After introducing a 100% leaflet dysfunction, the majority of cases exceeded 3 m/s (67%).

Figure 9B shows the relationship between LVOT diameter, percentage of dysfunction and mean transvalvular pressure gradient (TPGmean). It was strongly flow-dependent and proportional to flow rate for all cases (the higher the flow rate, the higher the TPGmean). Furthermore, TPGmean was strongly proportional to the severity of subaortic stenosis for all cases (the smaller the LVOT diameter, the higher the TPGmean).

For all cases of normally functioning BMHVs, TPG_mean_ did not exceed the TPGmean magnitude of 20 mmHg, regardless of subaortic stenosis severity (the suggested ASE threshold for possible dysfunction). Meanwhile, in cases of 50% leaflet dysfunction, the TPG_mean_ for only one case at the high flow rate (7 L/min) exceeded the 20 mmHg thresholds (7%). After introducing a 100% leaflet dysfunction, almost half of the cases exceeded the 20 mmHg thresholds (47%), and the highest TPG_mean_ magnitude was 61 mmHg. It is worth noting that none of the 0% and 50% dysfunction cases and only three cases of 100% leaflet dysfunction exceeded the higher limit of ASE threshold (35 mmHg).

The severity of subaortic stenosis had a significant impact on the elevation of the peak velocity and TPG_mean_ for dysfunctional BMHVs. Peak velocity and TPG_mean_ were increased by 39% and 107%, respectively, by reducing the LVOT diameter to 19 mm.

#### 3.2.2. Doppler Velocity Index (DVI)

Figure 9C shows the relationship between LVOT diameter, percentage of leaflet dysfunction and DVI. DVI magnitude was flow-independent. However, DVI magnitude was inversely proportional to the severity of subaortic stenosis for all cases (the smaller the LVOT diameter, the higher the DVI magnitude). On the contrary, DVI magnitude was inversely proportional to the percentage of BMHV dysfunction for all cases (the higher percentage of leaflet dysfunction, the lower the DVI magnitude).

For all cases of 0% and 50% leaflet dysfunction BMHVs, DVI did not fall below the DVI magnitude of 0.3 regardless of subaortic stenosis severity (the suggested ASE threshold for possible dysfunction). Meanwhile, in the cases of 100% leaflet dysfunction, DVI for eight cases fell below the 0.3 threshold (53%). Moreover, the lowest DVI magnitude did not fall below 0.25 (the suggested ASE threshold for significant dysfunction).

#### 3.2.3. Effective Orifice Area (EOA)

Figure 9D shows the relationship between LVOT diameter, percentage of leaflet dysfunction and EOA. EOA magnitude was flow-independent. However, EOA magnitude was strongly proportional to the severity of subaortic stenosis for all cases (the smaller the LVOT diameter, the smaller the EOA magnitude). On the contrary, EOA magnitude was inversely proportional to the percentage of BMHV dysfunction for all cases (the higher the percentage of leaflet dysfunction, the lower the EOA magnitude).

For all cases of 0% and 50% leaflet dysfunction BMHVs, EOA did not fall below the EOA magnitude of 1.2 cm^2^ regardless of subaortic stenosis severity (the suggested ASE threshold for possible dysfunction). Meanwhile, in cases of 100% leaflet dysfunction, EOA for 11 cases fell below the 1.2 cm^2^ threshold (73%). Moreover, the lowest EOA magnitude did not fall below 0.8 cm^2^ (the suggested ASE threshold for significant dysfunction).

Finally, Table 1 shows the relationship between LVOT diameter and Doppler echocardiographic parameters. Peak velocity, TPGmean and DVI are inversely proportional to LVOT size, and only EOA is proportional to LVOT size. The values of peak velocity ranged between 0.86 m/s and 6.20 m/s, the values of TPGmean ranged between 1.4 mmHg and 60 mmHg, and the values of DVI ranged between 0.25 and 0.89. However, the values of EOA ranged between 0.86 cm^2^ and 2.87 cm^2^.

### 3.3. Sensitivity and Specificity Analysis for Different Echo Doppler Parameters

Sensitivity and specificity analysis were conducted for conventionally used non-invasive Doppler echocardiographic parameters (Table 2). All the cases were categorized into two groups based on the level of dysfunction. The first group included both levels of severity (50% and 100% dysfunctional leaflet), which represents mild to severe BMHV dysfunction, while the second group included only the 100% dysfunctional leaflet, which represents moderate to severe BMHV dysfunction.

All the listed parameters had a relatively high specificity for the diagnosis of the dysfunction of prosthetic heart valves, and the lowest specificity value was for peak velocity (Vpeak≥3 m/s) (90%). However, all ASE-suggested criteria had a relatively low sensitivity in detecting the dysfunction of BMHV; EOA ≤ 0.8 cm^2^ criterion showed the lowest sensitivity (0%), and Vmax ≥ 3 m/s criterion showed the highest sensitivity (73%).

## 4. Discussion

### 4.1. Flow Characteristics

In this study, turbulence characteristics, in terms of velocity magnitude and direction (inertial force), flow separation, vorticity distribution and turbulent shear stress, were investigated, and the coexistence of subaortic stenosis with a dysfunctional heart valve altered the fluid dynamics, as follows:

(1) Velocity jets: introducing BMHV dysfunction reduced the EOA and changed the arrangement of the flow from a three-jet flow to having mainly one dominant stronger lateral jet. Consequently, introducing the subaortic stenosis increased the strength of the velocity jet/s and increased the traveled distance of the flow jet.

(2) Recirculation zones: the above-mentioned dramatic increase in velocity was accompanied by profound shear layers on the valve’s leaflets, followed by flow separation and Von Karman vortex Street shedding in the wake of the leaflets. Regardless of the level of disease severity, the conventional aortic sinus vortex was available and did not relocate itself, but instead the strength and the size of the vortex was proportional to the level of both valve dysfunction and subaortic stenosis. Valve dysfunction and subaortic stenosis were strongly associated with a greater presence of recirculation zones downstream of the BMHV, and also were associated with late dissipation for such zones [45].

(3) Turbulent shear stress: a dramatic increase in velocity gradient, due to flow separation in the vicinity of the leaflets and the formed recirculation zones, elevated TSS magnitude (almost 10-fold, compared to the normal case) and also increased the regions with relatively high turbulent shear stress [ref]. Interestingly, the maximum magnitude of TSS was found in the contact line between the velocity jet and the recirculation zone in the sinus area [39].

The coexistence of subaortic stenosis and BMHV dysfunction disturbed the blood flow and significantly increased the turbulence intensity, as a result of magnified velocity magnitude and the remarkable increase in the strength, size and number of recirculation zones [46].

### 4.2. Platelet Activation and Blood Components Damage

The presence of sole subaortic stenosis elevated the peak velocity through BMHV, which in turn amplified the magnitude of TSS. Adding to that, the concomitant presence of both subaortic stenosis and BMHV dysfunction led to an additional increase in peak velocity and TSS magnitudes. (TSS increased up to three-fold, and peak velocity increased up to two-fold compared to the cases of only dysfunctional BMHVs). Moreover, the strength of the vortical structures of blood flow downstream of the valve was significantly greater in the concomitant presence of subaortic stenosis with BMHV dysfunction, and the potential of residential time of blood components downstream of the valve were relatively higher.

The non-physiological vortical flow, with the high velocity and turbulent shear stress that was mainly introduced by the concomitant presence of the dysfunctional mechanical valve, might encourage the coagulation process and form a thrombus. Activated platelets with long residential time in the recirculation regions may aggregate, leading to free emboli formation [9].

### 4.3. Evaluation of Doppler Parameters

The subaortic stenosis might worsen the severity of valve dysfunction by magnifying the values of Vpeak and TPGmean, and reducing the values of EOA. On the other hand, the presence of subaortic stenosis might increase the values of DVI, and mask the probable valve dysfunction. Reducing the LVOT diameter (from 27 mm to 19 mm) played a major role in increasing the Vpeak value from 1.33 m/s to 1.81 m/s (~36% increase), the TPGmean value from 3.3 mmHg to 5.4 mmHg (~64% increase), and the DVI value from 0.6 to 0.9 (50% increase) for the case of a normally functioning valve at the normal cardiac output (5 L/min). Moreover, and for the same flow rate condition, the EOA value decreased from 2.7 cm2 to 2.1 cm^2^ (22% reduction).

It is also worth noting that the Vpeak and TPGmean are flow-dependent parameters. For the isolated 100% leaflet dysfunction at different flow rates, the Vpeak value increased from 0.84 m/s to 1.94 m/s (127% increase), and the TPGmean value increased from 1.42 mmHg to 6.47 mmHg (350% increase). Moreover, the DVI and EOA were shown to be the less flow-dependent parameters [19,36].

BMHV dysfunction may advance quickly from mild to severe dysfunction, and may thus become fatal within a short period. Therefore, the accurate diagnosis of valve dysfunction at an early stage is essential to promptly starting treatment (thrombolysis or surgery) before the case worsens. However, since all the Doppler parameters have suboptimal sensitivity for the detection of mild BMV dysfunction, it is useful to evaluate valve leaflet motion, via cinefluoroscopy, when there is a fear of BMHV dysfunction during the clinical or echocardiographic exam.

Finally, despite the fact that both DVI and EOA are less flow-dependent, the influence of subaortic stenosis was significant. Therefore, using Doppler parameters to evaluate BMHV performance without considering the severity of subaortic stenosis and the cardiac output will jeopardize the accuracy of the evaluation.

## 5. Conclusions

In the present study, the flow patterns resulting from the concomitant presence of subaortic stenosis with dysfunctional BMHV under unsteady-state conditions is investigated. The study demonstrates the significant impact of SAS on hemodynamics, in terms of elevating the magnitudes of velocity, turbulent shear stress and vorticity. The study shows that the blood flow is characterized by a cascade of vorticities, especially during the deceleration phase of the systole, and by markedly elevated turbulent shear stress, which in turn increases the chance of platelet activation and/or hemolysis.

The study suggests that, in the presence of subaortic stenosis, the accuracy of current Doppler echocardiographic parameters and criteria is jeopardized, especially in the detection of moderate-to-severe aortic BMV dysfunction. Among all addressed Echo-Doppler parameters, the following parameters show better sensitivity: DVI ≤ 0.35, Vpeak ≥ 3.0 m/s, and EOA ≤1.2 cm^2^, with corresponding values of 80%, 73% and 67%, respectively. Therefore, the study emphasizes the importance of the consideration of LVOT size during BMHV diagnosis.

Finally, additional in vitro and in vivo studies into dysfunctional BMHVs with the concomitant presence of subvalvular and supravalvular stenosis (altered geometry) have to be done to give a clear idea about the blood hemodynamics, and to improve the accuracy of conventional diagnostic methods and parameters.

## Figures and Tables

**Table 1:** Doppler echocardiographic parameters (range) as a function of LVOT diameter for different flow rates and deferent severities of BMHV dysfunctions.

Doppler Echocardiographic Parameter	LVOT Diameter		
	27 mm	23 mm	19 mm
**Peak Velocity (m/s)**	2.17 ± 1.09 (0.86–4.58)	2.45 ± 1.17 (0.91–4.89)	3.02 ± 1.47 (1.19–6.20)
**TGPmean (mmHg)**	9.03 ± 7.97 (1.42–28.71)	11.73 ± 10.46 (1.71–37.40)	17.68 ± 17.10 (2.44–60.53)
**Doppler Velocity Index (DVI)**	0.43 ± 0.14 (0.25–0.61)	0.53 ± 0.18 (0.28–0.78)	0.63 ± 0.21 (0.35–0.89)
**EOAmean (cm^2^)**	2.03 ± 0.59 (1.17–2.87)	2.14 ± 0.37 (1.66–2.63)	1.52 ± 0.49 (0.86–2.20)

The results expressed as mean ± SD and the interval represents the minimum and the maximum values.

**Table 2:** Sensitivity and specificity of Doppler parameters for all cases (with and without LVOT obstruction).

Parameter	Diagnostic Criteria for Dysfunction	Detection Mild-to-Severe Dysfunction (both 50 % and 100 % Dysfunctional Leaflets are Considered)		Detection Moderate-to-Severe Dysfunction (only 100% Dysfunctional Leaflet)	
		Sensitivity (%)	Specificity (%)	Sensitivity (%)	Specificity (%)
**Peak velocity**	≥4 m/s	20	100	40	100
	≥3 m/s	47	100	73	90
**TGPmean**	≥35 mmHg	10	100	20	100
	≥20 mmHg	27	100	47	97
**Doppler Velocity Index (DVI)**	≤0.35	40	100	80	100
	≤0.30	27	100	53	100
	≤0.25	3	100	7	100
**EOAmean**	≤1.2 cm^2^	33	100	67	100
	≤0.8 cm^2^	0	100	0	100
